# Supplementary figures and images for: Automated detection and prediction of suicidal behavior from clinical notes using deep learning
Source: PLoS One. 2025 Sep 15;20(9):e0331459. doi: 10.1371/journal.pone.0331459 (PMC12435685; doi:10.1371/journal.pone.0331459)

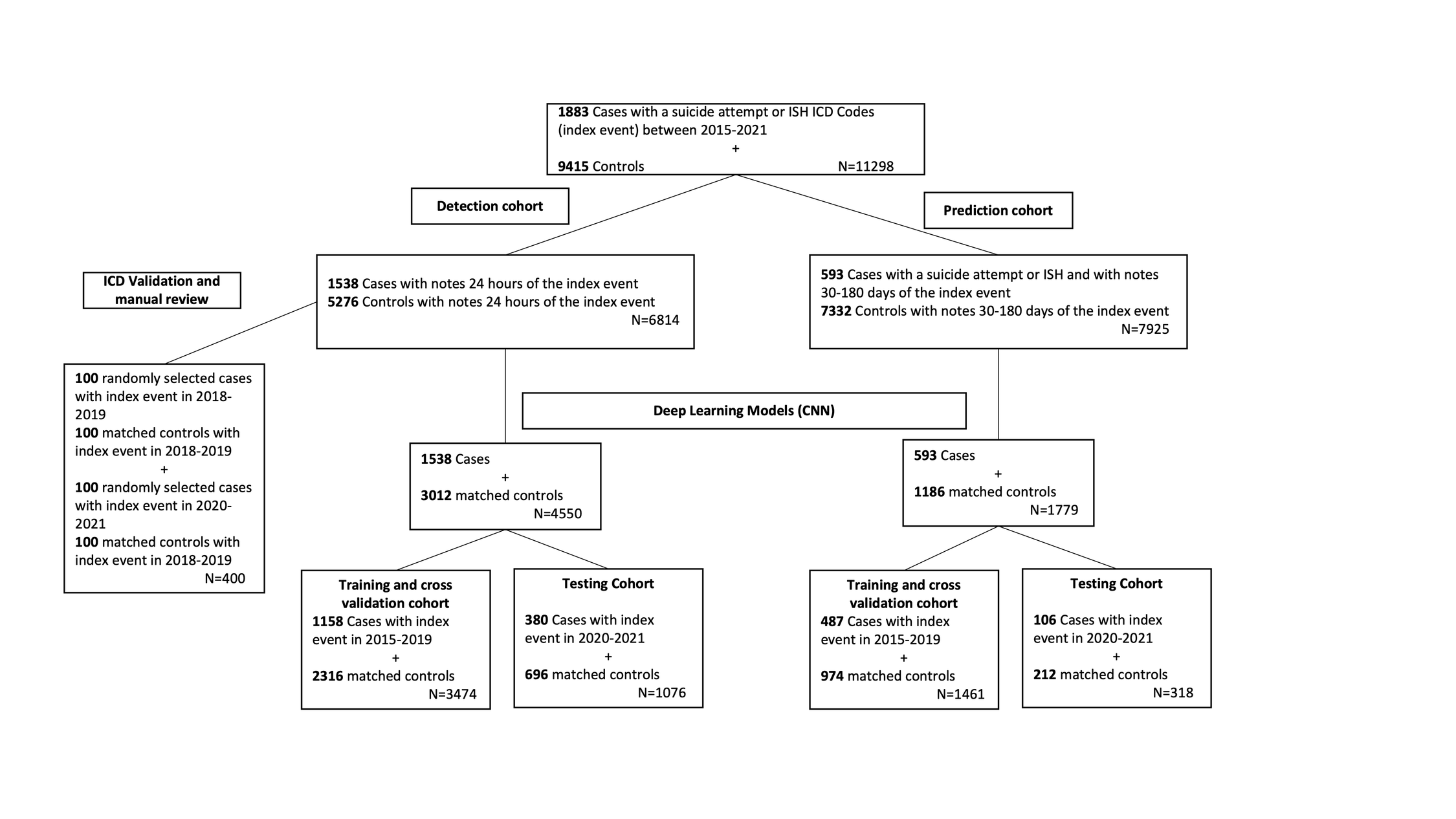

Supplement: S1 Fig — (TIF) [file pone.0331459.s001.tif]

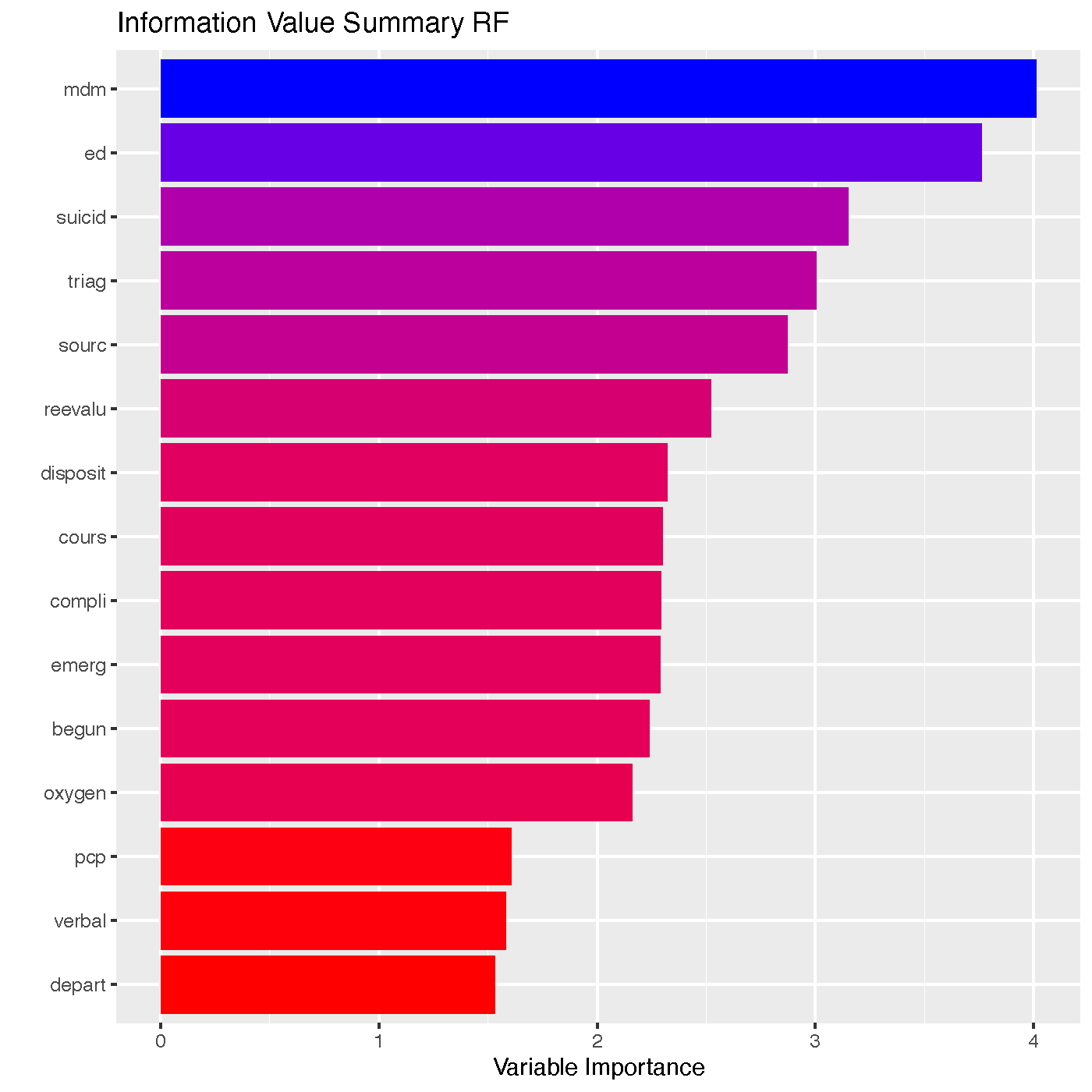

Supplement: S2 Fig — (TIF) [file pone.0331459.s002.tif]
