# Supplementary material for: Automated detection and prediction of suicidal behavior from clinical notes using deep learning
Source: PLoS One. 2025 Sep 15;20(9):e0331459. doi: 10.1371/journal.pone.0331459 (PMC12435685; doi:10.1371/journal.pone.0331459)
Supplement: S1 Table — (DOCX) [file pone.0331459.s003.docx]

S1 Table. Clinical note types and frequency per group

|  | Detection Cohort | | Prediction Cohort | |
| --- | --- | --- | --- | --- |
|  | Case (N=1,538) | Control (N=3,012) | Case (N=593) | Control (N=1,186) |
| Anesthesia Pre-Procedure Evaluation | 77 | 28 | 113 | 307 |
| Case Management | 508 | 9 | 267 | 79 |
| Consults | 707 | 102 | 1,040 | 1,333 |
| Discharge Patient Education Summary | 19 | 66 | 237 | 383 |
| ED AVS Snapshot | 90 | 30 | 205 | 67 |
| ED Notes | 6,560 | 318 | 2,417 | 990 |
| ED Provider Notes | 609 | 100 | 661 | 377 |
| ED Triage Notes | 282 | 44 | 127 | 88 |
| H&P | 564 | 79 | 354 | 605 |
| Incoming Trauma/Burn Note | 228 | 0 | 7 | 4 |
| Interdisciplinary Plan of Care Note | 424 | 79 | 865 | 918 |
| IP AVS Snapshot | 10 | 30 | 203 | 261 |
| Op Note | 40 | 25 | 88 | 256 |
| OR Nursing | 168 | 59 | 103 | 507 |
| Plan of Care | 1,724 | 444 | 4,527 | 5,622 |
| Poison Center Initial Note | 27 | 0 | 4 | 0 |
| Procedure | 101 | 64 | 214 | 366 |
| Progress Note | 1,151 | 2,433 | 5,797 | 11,639 |
| Psychiatry Note | 5 | 7 | 47 | 24 |
| Undefined | 4,523 | 2,067 | 4,326 | 6,583 |
| Total number of notes | 17,817 | 5,984 | 21,602 | 30,409 |
| Average number of notes per person | 11.6 | 1.98 | 36.4 | 163.5 |
