# Supplementary material for: Automated detection and prediction of suicidal behavior from clinical notes using deep learning
Source: PLoS One. 2025 Sep 15;20(9):e0331459. doi: 10.1371/journal.pone.0331459 (PMC12435685; doi:10.1371/journal.pone.0331459)
Supplement: S2 Table — (DOCX) [file pone.0331459.s004.docx]

| S2 Table. Participant Demographics | | | | | | |
| --- | --- | --- | --- | --- | --- | --- |
|  | Detection Cohort | | | Prediction Cohort | | |
|  | Case  *n* = 1,538 | Control  *n* = 3,012 | *p* | Case  *n* = 593 | Control  *n* = 1,186 | *p* |
|  | M (SD) | M (SD) |  | M (SD) | M (SD) |  |
| Age | 39.0 (15.4) | 45.4 (16.2) | .001 | 42.0 (16.2) | 42.15 (16.0) | .826 |
|  | n (%) | n (%) |  | n (%) | n (%) |  |
| Sex |  |  | .001 |  |  | .207 |
| Female | 692 (45%) | 1,881 (62%) |  | 340 (57%) | 717 (60%) |  |
| Race |  | | .921 |  | | .937 |
| White | 990 (64%) | 1,931 (64%) |  | 401 (67%) | 812 (69%) |  |
| Black/AA | 245 (16%) | 473 (16%) |  | 99 (17%) | 193 (16%) |  |
| Other | 303 (20%) | 608 (20%) |  | 93 (16%) | 181 (15%) |  |
| Ethnicity |  | | .110 |  | | .961 |
| Hispanic/Latino | 246 (16%) | 482 (16%) |  | 91 (15%) | 176 (15%) |  |
| Non-Hispanic/Latino | 1,249 (81%) | 2,409 (80%) |  | 499 (84%) | 1,004 (85%) |  |
| Other | 43 (3%) | 121 (4%) |  | 3 (1%) | 6 (0%) |  |
