# Supplementary material for: Automated detection and prediction of suicidal behavior from clinical notes using deep learning
Source: PLoS One. 2025 Sep 15;20(9):e0331459. doi: 10.1371/journal.pone.0331459 (PMC12435685; doi:10.1371/journal.pone.0331459)
Supplement: Attachment — Submitted filename: Response to Reviewers.docx [file pone.0331459.s005.docx]

Dear PLOS ONE Editor,

Thank you for your response to our submission entitled “Automated detection and prediction of suicidal behavior from clinical notes using deep learning” [PONE-D-24-31813] - [EMID:2dd056c362e38b9e]. We would like to thank the reviewers for their insightful critiques and comments. We believe that we have addressed their concerns and that the revised manuscript is much improved. Below are point-by-point responses to the comments, along with descriptions of our modifications in the revised manuscript.

# Reviewer #1

**REVIEWER COMMENT:** The authors need to more explicitly articulate why and how they demonstrate the generalizability of the methodology they apply. I appreciate that the authors' objective is to reproduce the methodology of Obeid et al (2020), but it is not clear why it would be a challenge to reproduce the methodology given the existence of ICD-coded data and access to notes. Why is it interesting to investigate the reproduction of this method? What can we learn from this attempt? The authors do not explain this point or truly "validate" the reproduction of the method, as they claim is the core objective of the paper (p. 5). Is it enough to follow the same methodology and present results of a model built from that methodology to "evaluate [the methodology's] generalizability to other academic medical centers? Given that -- as the authors state -- the method hinges on the availability and quality of ICD coded records, that seems to be the only point. Or is it that a certain expected level of performance of the model must be achieved to claim that the methodology is generalizable? It simply isn't clear what the criteria for "validating" the generalizability of the methodology are. How can we claim success on this goal?

**AUTHOR RESPONSE:** We thank the reviewer for this comment, and we hope to clarify this point. Before this work, there was no documentation in the literature other than from Obeid et al. 2020 about 1) the accuracy of ICDs in this domain and 2) the utility of ICDs as “silver standard” labels to train models. We strongly believe that reproducibility in other settings is important since it is not enough to show that a method is generalizable if it works at only one institution. In this manuscript, we have shown that both these points are true at another academic medical center. Overall, we achieved comparable levels of performance at both institutions. Specifically, the AUC of the CNNr model in Obeid et al. 2020 was 0.882 and in this study, it was 0.817, while the AUC of CNNw in Obeid et al. 2020 was 0.869 and in this study was, 0.812. These AUC values indicate models of outstanding performance. Moreover, we have shown that local context may impact the prediction. In this study, we have shown that phrases specific to local practices (e.g., the Baker Act) may impact the risk probability. We have updated the text to emphasize these points.

**REVIEWER COMMENT:** The real-world applicability of the method is still not adequately addressed/discussed given artificial selection criteria when constructing the cohorts. More importantly, the significance of the original methodology and the new application of that methodology in this new setting is unclear.

**AUTHOR RESPONSE:** We thank the reviewer for their attention to this. As noted in our response to the previous comment, the significance of this and the original methodology are 1) the accuracy of ICDs in this domain and 2) the utility of ICDs as “silver standard” labels to train NLP models. Further, the replication of the prior methodology is significant as this is a core tenant of the scientific method. With respect to real-world applicability, this work helps to establish a foundation for the future development of tools designed to detect ISH in real-world clinical settings, which we have outlined in the Discussion section.

**REVIEWER COMMENT:** As the authors themselves point out, it is more important to examine how well a trained model itself generalizes -- both to more real-world settings and to other hospital sites. This is not tackled by the authors of this work.

**AUTHOR RESPONSE:** We agree wholeheartedly with the reviewer. Unfortunately, sharing models across institutions was outside the scope of this project’s funding, and we have now emphasized this in the Limitations section.

**REVIEWER COMMENT:** As I will mention below, the datasets used to train and test the authors' model do not appear to be fully realistic. The test data in particular would be much more interesting if it reflected the full complexity of the real-world data environment, including the low prevalence of ISH presentations overall.

**AUTHOR RESPONSE:** The reviewer brings up a valid and important point. A large enough dataset of ISH cases is required to train the NLP models. However, at a single institution, we do not have sufficient data to develop and test the models in a real-world environment. We agree with the reviewer, and we have added this to the paper’s limitations and future directions.

**REVIEWER COMMENT:** I grant that the performance of the models is well beyond random chance, but overall it seems that there is significant uncertainty as to how well this model would work prospectively in the natural data environment, given the steps taken to artificially ensure a minimum number of Cases in the datasets (at least 1/3), while also not controlling for Controls that are the most similar to the Cases. Controls are "matched" only in terms of a superficial note profile, plus to control numbers, not to identify the most likely potential confuses for the model.

**AUTHOR RESPONSE:** The reviewer is correct that we matched on criteria that may potentially bias predictions based on the characteristics of the population (e.g., gender and race) and artificial biases (such as the number of notes per patient). We believe this approach retains the rest of the clinical content in notes, including the patient’s present illness and history of illness, to assess its value in phenotyping and predicting ISH. Our artificial cut-off of the population (namely 3 to 1) was a pragmatic approach to balance between the minimum necessary records needed to conduct this work and the ability to reduce bias by matching.

**REVIEWER COMMENT:** When constructing the cohort datasets, did you ensure that the same patients did not appear in both training and test data sets? i.e. not simply splitting at the event level, but rather at the patient level. This is important to control for data leakage.

**AUTHOR RESPONSE:** We thank the reviewer for their attention to this. To clarify, we ensured that there was no data leakage; the data portioning was done at the patient level and not the individual note level.

**REVIEWER COMMENT:** The data also appears to include "ED After Visit Summary (AVS)" reports, which I suppose are something like a Discharge Summary. Given that this is very likely to have a complete review/summary at the end or after of an ED episode, it seems that this type of report should be excluded as it will not be relevant to detecting a patient with ISH while they are in care.

**AUTHOR RESPONSE:** We tested both the phenotyping and the predictive power of historical notes. In the phenotyping component, the objective was to show that such models could be used for automated surveillance to identify patients with ISH, whereas, with historical notes, the notes were selected 1 month or more prior to the presentation with ISH, so all components of the discharge summary were documents before the presentation of ISH and are therefore fair game for predictions.

**REVIEWER COMMENT:** Why was a 1:5 ratio adopted for Cases:Controls? To what extent is this representative of the overall prevalence of ISH in the data? Then, why was a 1:2 ratio assumed for "matched controls" in the final training/testing cohorts? This results in training/test cohorts that are very far out of balance from the a priori probability of ISH in the overall dataset.

**AUTHOR RESPONSE:** We appreciate the opportunity to clarify this point. We extracted 5 controls per case from the EHR to allow for better cohort matching. Then, we matched 2 controls for every case used (2:1 ratio controls to cases) to create the cohorts for the development/testing of the models. This has been clarified in the Methods section.

**REVIEWER COMMENT:** I assume that "Controls" are patients that have no (0) suicide attempts or ISH ICD Codes at all in the data, although this is not fully specified. Then you filter out Controls that meets the notes requirements.

**AUTHOR RESPONSE:** The reviewer is correct. Controls are patients who have no suicide attempts or ISH ICD codes at all in the data. We have clarified this point in the Methods section.

**REVIEWER COMMENT:** What is the "index event" that is considered for a Control?

**AUTHOR RESPONSE:** The index event for cases was defined as the first suicide attempt or ISH diagnosis, and the index event for controls included the most recent interaction within the EHR. We have clarified this point in the Methods section.

**REVIEWER COMMENT:** Significant differences in the types of clinical texts are reported for cases vs controls in both the Detection and Prediction cohorts. This arguably means that distinguishing between cases and controls is easier; it is not only the content of the notes that varies, but the a priori nature of the information they reflect. These differences also imply substantive differences in the nature of problems and patterns of care that the cases and controls may be attended for. There would presumably be much more extra-cases variability, than intra-cases variability, which again makes distinguishing between them potentially very easy.

**AUTHOR RESPONSE:** We should note, however, that the variable importance analysis of the RF model, in both the detection of concurrent events and the predictive models, provided some insight into important features (characteristics within the content of the clinical notes) that are indicative of ISH. Important variables included stemmed words such as “suicid”, “attempt” and “ed”, as well as words that are relevant only in the local context, such as “ba” as in the Baker Act, which are specific to Florida. In our experience, this is evidence that the models are prioritizing the content of the notes over the structure of the notes or note types. We have included this discussion in the manuscript.

**REVIEWER COMMENT:** What is the reason for Cases having so many more notes than Controls (11.6 vs 1.98) -- is this because there are many (short) ED notes cf. fewer (long) Progress Notes for Controls?

**AUTHOR RESPONSE:** We agree with the reviewer that this was likely due to the increased number of notes typically entered by various specialties within the 24 hours prior to an Emergency Department visit *vs*. the fewer number of notes associated with general healthcare visits.

**REVIEWER COMMENT:** Given the predominance of ED notes for Cases cf Controls, why was a "matched control" not one that had a similar notes profile to a Case, in terms of type of notes, number of notes, length?

**AUTHOR RESPONSE:** The reason for not having similar note profiles between cases and matched controls is because we used the nearest neighbor matching algorithm, which prioritizes cases based on proximity in selected characteristics rather than aiming to create an identical profile for all features, such as type, number, and length of notes.

**REVIEWER COMMENT:** It is not fully clear what data the manual review of clinical notes includes -- does this mean up to 8000 words of text, as is prepared for the model? Or full chart review?

**AUTHOR RESPONSE:** We are happy to clarify this for the reviewer. The manual review included concatenated notes truncated to 8000 words of text in a similar manner as the notes prepared for the model. There was no full chart review. We have noted this in the revised manuscript.

**REVIEWER COMMENT:** It is one thing to *train* supervised learning models with "silver standard" data. It is another to *test* the models on silver standard data. What did you do to ensure the reliability of the final results on the held-out test data?

**AUTHOR RESPONSE:** We agree with the reviewer on this point. To ensure the reliability of the final results, we assessed the performance of the CNNr model on the smaller 400 records dataset used for manual review. We have appended Table 1 with the performance of this model.

# Reviewer #2

**REVIEWER COMMENT:** In this manuscript the authors posit that deep learning approaches have great potential to significantly improve traditional suicide prediction models to detect and predict intentional self-harm and report that existing research is limited by a general lack of “consistent performance” and “external reproducibility”. The work in the paper is aimed to “validate a deep learning approach” used in previous research [reference 27] to detect and predict ISH using clinical note text and “evaluate its generalizability to other academic medical centers”.

The manuscript is well written and properly structured without significant obstacles to understandability in terms of language and format. The methodology is sound and well explained though the methodology is not the novel contribution of the manuscript and has been published and implemented in [27]. Therefore, as I understand it, the main contribution of this paper is applying this modeling process (methodology) to another data set also validated by the statement from the manuscript “Thus, the purpose of the current study was to validate the approach used previously by Obeid et al. (2020) and to evaluate its generalizability to other academic medical centers.” (page 5 of manuscript). In the limitations section the authors indicate “This study has limitations that provide opportunities for future research. First, although we were able to reproduce the original work conducted by Obeid and colleagues (2020) at an additional medical center, it is necessary to show reproducibility across multiple sites. Ideally, we would also want to perform external validation of a trained model (i.e., train at one institution and test the trained model at another site).

**AUTHOR RESPONSE:** We thank the reviewer for the positive feedback and suggestions for improving our manuscript.

**REVIEWER COMMENT:** The authors should go through the manuscript and clarify the distinction between model and process (methodology) validation and specify that the contribution of the paper is to process validation and not model validation.

**AUTHOR RESPONSE:** We thank the reviewer for this suggestion. We have reviewed and revised the terminology as suggested.

**REVIEWER COMMENT:** The authors indicate that lack of consistent performance is an important limitation of existing research. While at least as far as process validation is concerned they are able produce consistent results, the authors are silent on what could be the potential cause of such variability. Is it the data used, the methodologies, a combination of the two? OR is it only normal that when such a wide net including structured EHR vs clinical note text with sub-populations such as those with eating-disorders, first time suicide attempts, etc. is considered, such variability is inevitable. I believe the authors possess the experience to address, or at a minimum discuss, the reason for this gap in existing research.

**AUTHOR RESPONSE:** We thank the reviewer for this suggestion. We agree that understanding the source of variability in performance across institutions is essential. We believe that this variability is influenced by a combination of factors such as differences in ICD coding practices, local context, and population characteristics. For example, in this study, we identified the words “Baker act” which is a Florida-specific term used when someone is in danger of harming themselves or others and which does not exist in the notes from the Obeid et al. 2020.

**REVIEWER COMMENT:** The audience would be greatly interested in understanding why a model validation (along with process validation) was not reported in the manuscript. Did the authors face difficulties or simply have not attempted it (to me it does not seem like a difficult extension)? As the authors themselves indicate, this is also very important, and the readers would appreciate their experience and perspective.

**AUTHOR RESPONSE:** We appreciate this comment and recognize the need for model validation between the institutions. However, this validation will be a part of future research due to funding limitations and the extensive regulatory hurdles associated with data sharing that cannot be fully de-identified.

**REVIEWER COMMENT:** The cited literature is until 2022 and should be updated as we are approaching 2025.

**AUTHOR RESPONSE:** We have updated the cited literature to included more recently published articles.

**REVIEWER COMMENT:** The term “concurrent ISH” is used several times in the manuscript. The reader is not clear on the meaning of the word “concurrent” here.

**AUTHOR RESPONSE:** We thank the reviewer for bringing this to our attention. We have now operationally defined “concurrent ISH” early on in the manuscript to help clarify this.

**REVIEWER COMMENT:** On page 11, the section on “Clinical Note Types and Frequencies” presents a number of results and it is unclear why these are f interest or how these impact the detection and/or the prediction process. Perhaps these should be discussed in the discussion section.

**AUTHOR RESPONSE:** We thank the reviewer for this suggestion. We have renamed the section “Clinical Note Characteristics”. We believe it is critical for the readership to understand, in addition to the patient characteristics, the characteristics of the notes used to build the models.

**REVIEWER COMMENT:** On page 14, “Although the use of pre-trained W2V word embeddings did not significantly improve the accuracy in the phenotyping task or exceed the accuracy using randomly initialized word embeddings in the predictive task, we have shown in previous unpublished work that W2V can result in faster training requiring fewer epochs during the training of classifiers. This effect was not evaluated in this study.” perhaps should be removed as it relies on unpublished work.

**AUTHOR RESPONSE:** We agree with the reviewer and have removed this statement from the manuscript.

# Reviewer #3

**REVIEWER COMMENT:** This research paper attempted to validate a previous deep learning approach they had developed to detect and predict intentional self-harm using clinical note text. A secondary aim was to assess a set of ICD codes as a silver standard for training models on the automated detection of intentional self-harm and to predict future encounters of ISH.

**AUTHOR RESPONSE:** We thank the reviewer for their suggestions for improving our manuscript.

**REVIEWER COMMENT:** The term “well-defined set” is vague. Is it the full list from Hedegaard, 2018?

**AUTHOR RESPONSE:** We appreciate the opportunity to clarify this. The full list of ICD codes from Hedegaard, 2018 was used to identify ISH. We are open to further suggestions from the reviewer on how this might be stated more clearly.

**REVIEWER COMMENT:** As stated in that same paper and then found extensively in the suicide NLP literature, ICD codes are very incomplete in detecting suicidality. Perhaps self-harm behavior and suicide attempt is a bit better than suicide ideation, but it is still known to have very low sensitivity for identifying cases, with less than half (and sometimes even much, much less than that) of cases detected by ICD codes. The authors’ manual validation of cases and controls as identified by ICD codes is in stark contrast to any literature known by this reviewer. That begs the question of why. Is it the underlying cohort of patients/corpus of notes? The paper does not specify the details of the underlying corpus of 1,538 and 3,012 cases and controls. Is this from the entire EHR in the specified date range? Perhaps the manual review for the controls was affected by ISH being a rare event so not enough notes were seen? Perhaps the control group should be enriched to get a better sense of the false-negative rate of ICD code detection.

**AUTHOR RESPONSE:** We appreciate this comment from the reviewer. We would like to begin by clarifying the process we followed for the manual validation: We extracted from the EHR all notes recorded within 24 hours of the index event for each patient. Each note was truncated to 800 words. A patient’s notes were then ordered chronologically and concatenated. The final string of the concatenated note was truncated to 8000 words.

This process was replicated in both institutions and yielded the results with similar accuracy. Specifically, in Obeid et al. (2020) the accuracy of the intentional self-harm ICD codes attributed to concurrent visits was 0.92, with a precision of 0.84 and recall of 1.0. That said, the reviewer is absolutely correct that we chose our controls randomly (aside from the matching mentioned within). Therefore, ISH, being rare, may not have been adequately represented to identify sufficient false negatives. The idea of enriching the data set with patients at high risk (e.g., history of depression) may improve the analysis of sensitivity; however, that would have to be generalized into the broader, not-at-risk population. In fact, in follow up research outside the scope of this manuscript, we are looking at this modeling within a high-risk population. We have added this as a future direction in the Discussion section of the manuscript.

**REVIEWER COMMENT:** Another thing that made this reviewer question the validity of the cases and controls was the supplemental material that indicated that many of the ISH encounter notes were from things like anesthesia pre-op OR nursing, and procedure notes. That seems quite odd that discussion and/or coding of self-harm would come up in this setting. During a surgical procedure? Or while the anesthesiologist is seeing the patient for 5 minutes to brief them? I would be curious about the strings that are being reviewed by the manual reviewers from these notes. Are the strings extracted out of order from the original note? Perhaps there is something that is causing a lot of false-positive mentions here… If one of the objectives of the paper is to validate the ICD list as a silver standard (which again goes against the literature), there needs to be more discussion here to establish credibility.

**AUTHOR RESPONSE:** We appreciate the opportunity to clarify this. We included all clinical notes recorded within 24 hours of the index event where an ISH ICD code was provided. Thus, not all of those notes were associated for the index event. For example, a patient could have presented in the ED with a self-inflicted gunshot wound and provided an ISH ICD code. Given that that patient would likely need surgery within that 24-hour period, anesthesia and pre-op notes occurring within 24 hours of the index diagnosis would be extremely likely. This does not mean that those notes would necessarily be labeled with the same ISH ICD code, but they would have been included, nonetheless.

**REVIEWER COMMENT:** If the determination is that ICD codes have precision of 0.8 and recall of 0.99 (which again seem suspect), those accuracy scores are much greater than the deep learning approaches. What then is the rationale for using the deep learning approach?

**AUTHOR RESPONSE:** We thank the reviewer for this comment. While ICD codes may exhibit high precision and recall in manual review, using a deep learning approach is advantageous for several reasons, including: capturing/utilizing unstructured information, examining generalizability across institutions where ICD coding practices may differ, complementary utilization of ICD code and deep learning (as a future application), and most importantly, potential real-time identification of ISH before ICD codes are recorded in the EHR.

**REVIEWER COMMENT:** Question regarding concurrent ISH: This reviewer is unclear if this means the ISH event occurred leading up to this encounter? That is what the text seems to imply. But it’s impossible to distinguish from it just being the first time that the ISH is known and documented, right? Like if it’s their first time seeing a psychiatrist and the psychiatrist asks about history of suicide attempts even though it happened 10 years ago? Or do you exclude historic mentions somehow?

**AUTHOR RESPONSE:** We appreciate the opportunity to clarify this. First, in response to a suggestion from another reviewer, we have provided a clearer operational definition for concurrent ISH early on in the manuscript. The index event in the ISH cohort was the first ISH diagnosis in the defined timeframe. The notes for concurrent ISH were extracted 24 hours prior to this index event. Notes from a suicide attempt that may have happened 10 years ago were not considered.

**REVIEWER COMMENT:** Along the same lines, how were negated mentions accounted for? Many clinicians might document the absence of ISH.

**AUTHOR RESPONSE:** We would like to note that this is a limitation of “bag-of-words” models that rely on instances of single words (unigrams). However, these models still evaluate the whole note by assessing the frequencies of other words relevant to ISH that increase the probability that a patient is, in fact, an ISH patient. Moreover, with deep learning models (i.e., CNNs), the sequence of the words (or tokens) is preserved, which has a greater potential of picking up negations. We have added this information in the discussion.

**REVIEWER COMMENT:** The justification sentence: “However, current approaches to suicide risk assessment involve healthcare professionals administering clinical interviews and questionnaires, which can be inefficient, costly, and limited in their ability to predict future ISH and suicide deaths.” This is circular logic since the notes themselves are documentation of the clinical interviews and assessments. We wouldn’t want clinicians to stop doing what they do—assessing patients. And also if the theoretically did that, what would the algorithm use to predict, since it is relying on mentions of “suicid” etc.?

**AUTHOR RESPONSE:** We thank the reviewer for this comment. We agree that clinical assessments are essential and do not suggest that clinicians should stop performing interviews or assessments. Instead, this is a justification for this line of work, not solely the project reported in this manuscript. This work using clinical note text for suicide surveillance and identifying suicide risk sets the stage for future work that will be more meaningful for real world data and clinical settings. This is more of a proof of concept that we can use text to effectively accomplish these tasks. Further, we envision that current approaches to suicide risk assessment can benefit from complementary, deep learning driven tools that utilize existing clinical documentation to provide additional and potentially valuable information. We have added text to the Introduction section that we hope further clarifies this point.

**REVIEWER COMMENT:** This implication in the Clinical Implications section makes a lot of sense and could be emphasized more including in the objectives: “This would be especially useful in cases where ISH risk is documented in prior clinical notes but is not added to a patient’s primary problem list, and therefore not easily viewable by providers who were not the original documenter.”

**AUTHOR RESPONSE:** We appreciate this suggestion and have emphasized this in our objectives.

**REVIEWER COMMENT:** Later on in the same section: “also the observation and adjustment of those risk levels over time” – not sure what that means.

**AUTHOR RESPONSE:** We appreciate this request for clarification. We have revised the sentence to read, “This would be useful for informing patient risk assessment–including quantifying risk levels–as well as the observation of those risk levels over time.”

**REVIEWER COMMENT:** In the limitations section: not sure what this means: “This highlights the need to examine this approach in the using data on fatalities resulting from suicide.”

**AUTHOR RESPONSE:** We thank the reviewer for pointing out this error. We have revised the sentence to read, “Moreover, although we demonstrated that we can clearly identify ISH, this does not specify an intention to die, so future work will need to examine this approach while incorporating data on fatalities resulting from suicide.”

**REVIEWER COMMENT:** This reviewer is not sure how similar/different this approach was to the original 2020 article from which it drew its methods. It would be helpful to know what has changed and what is different. And why the authors chose to continue these now outdated techniques and to not use language models. Not necessarily saying language models are always better, but would be an important discussion.

**AUTHOR RESPONSE:** We thank the reviewer for the opportunity for clarification. Our aim in this work was to show reproducibility of the approach of the original work, which was done at an entirely different institution. For example, we were able to show that the combination of ICD codes for ISH was fairly performant in picking up ISH at both institutions (with the limitations noted in a previous response), and that machine learning models, especially deep learning models, can be trained to identify ISH within clinical notes at different sites. However, we appreciate the reviewer’s comment and agree that newer models such as large language models should be included in the next phase in this work and have added this to our Discussion section. Nevertheless, as the reviewer suggests, there is still value in statistical machine learning models. Finally, another notable difference in this work from the cited work is that we had almost twice as many patients reviewed.

**REVIEWER COMMENT:** Finally, the 0.61-0.64 F1 scores are not so good… What does that mean about how to interpret this work and future

**AUTHOR RESPONSE:** We thank the reviewer for pointing this out. Although the F1 score seems low, the performance of the predictive model (especially as highlighted with the AUC) is fairly competitive with what has been previously reported in the literature using different approaches with EHR data, including structured data. This is important, because one can calibrate the threshold to improve recall/sensitivity vs. the specificity depending on the setting. For example, to improve the recall, we could reduce the threshold to pick up more cases for referrals. The high AUC suggests that we are more likely to find a good balance for a given use case or environment in which such models could be used. In critical applications like suicide prediction, maximizing recall is critical. By lowering the probability threshold, you increase the likelihood of identifying most patients at risk, even if it means having more false positives. We should note, however, that it is also crucial to continuously evaluate and refine the models, incorporating feedback from healthcare professionals and data from real-world outcomes to improve its accuracy and effectiveness over time. We have added this information in the discussion.

We would like to thank the reviewers for their diligent critiques and thoughtful comments. We hope that we have addressed the reviewers’ comments and suggested edits. We believe these edits have strengthened our submission. We are excited to share this work with the Frontiers in Psychiatry readership and the rest of the mental health research community.
