# Supplementary material for: Automated detection and prediction of suicidal behavior from clinical notes using deep learning
Source: PLoS One. 2025 Sep 15;20(9):e0331459. doi: 10.1371/journal.pone.0331459 (PMC12435685; doi:10.1371/journal.pone.0331459)
Supplement: Attachment — Submitted filename: R2 Response to Reviewers.docx [file pone.0331459.s006.docx]

Dear Editor,

Thank you for your response to our submission entitled “Automated detection and prediction of suicidal behavior from clinical notes using deep learning” PONE-D-24-31813R1]. We would like to thank the reviewers for their insightful critiques and comments. We believe that we have addressed their concerns and that the revised manuscript is much improved. Below are point-by-point responses to the comments, along with descriptions of our modifications in the revised manuscript.

# REVIEWER #1

**REVIEWER COMMENT:** Overall re-review: While I appreciate the authors' response to the previous round of reviews and the corresponding revisions to the paper, I still have some concerns about the overall significance of the study in relation to the claims that are being made. A stronger emphasis on process replicability cf model replicability is required, and more discussion of the limitations of the process and particularly the data selection methodology is required. i do agree that it is valuable to explore the application of the process in a new data context, but claims related to the "high AUC" should be moderated given the unnatural data distribution adopted for evaluation.

**AUTHOR RESPONSE:** We thank the reviewer for this insightful comment and for recommending the paper by Beam, Manrai, and Ghassemi (2020), which provided valuable guidance in refining our terminology and framing. In response, we have revised the manuscript to more accurately describe our work as an effort in replication rather than reproducibility, consistent with the distinctions outlined in that paper.

As Beam et al. clarify, reproducibility refers to the ability to obtain the same results using the same data and code, while replication involves applying the same methods to new data to assess whether similar conclusions can be drawn. Our study aligns with the latter: we applied a previously validated deep learning pipeline to a new institutional dataset to evaluate whether the approach generalizes across settings. Accordingly, we have replaced mentions of “reproducibility” with more precise language describing our work as a replication of the methodological pipeline on new data and added a reference to Beam et al 2020 (<https://doi.org/10.1001/jama.2019.20866>).

Regarding the unnatural data distribution, while this approach was necessary to ensure sufficient statistical power for model training and evaluation, we acknowledge that it introduces an artificial class balance that does not reflect the natural prevalence of ISH in the population. We now explicitly mention that performance metrics such as AUC may be inflated under these conditions in the limitations section.

**[Original] REVIEWER COMMENT:** Given the predominance of ED notes for Cases cf Controls, why was a "matched control" not one that had a similar notes profile to a Case, in terms of type of notes, number of notes, length?

**[Original] AUTHOR RESPONSE:** The reason for not having similar note profiles between cases and matched controls is because we used the nearest neighbor matching algorithm, which prioritizes cases based on proximity in selected characteristics rather than aiming to create an identical profile for all features, such as type, number, and length of notes.

**[New] REVIEWER COMMENT:** I find that this is not satisfactorily addressed. The differences in data between cases and matched controls would seem to bias the model, i.e. making it easier to detect the cases based on arguably spurious factors. I understand that the characteristics that were selected include "age, gender, race, ethnicity, and number of notes" -- so number of notes is considered, but only as one of 5 factors, of which 3 are categorical demographic features and therefore have less variance (so will dominate the matching). It would have been more interesting to identify controls that are more likely to be confusers, e.g. those with ICDs for other non-ISH psychological presentations, or as previously suggested that align more strongly in terms of the types of note -- noting the substantial differences between cases and controls described in the section "Clinical Note Characteristics". Clearly if the cases and controls have very different types of notes then distinguishing them shouldn't be very difficult for the model.

In general, more error analysis would help to elucidate what the model is actually learning. For example, for the "39 were diagnosed with ICD codes for suicide attempts or ISH when it was not documented in their clinical notes (i.e., False Positives)" and the "2 patients were not assigned ICD codes for suicide attempts or ISH when their clinical notes indicated they should have (i.e., False Negatives)." -- what was the performance of the model on these cases? Did the models also get confused by the FP cases, and did they detect the FN cases?

**AUTHOR RESPONSE:** We agree with the reviewer about the differences in note types between cases and controls. Ideally, those should have been included in the matching criteria, regardless of the possibility that they may reduce the numbers of controls further. So, although it is a good idea and should be considered in future experiments, it may further artificially inflate the ratio of cases to controls. Further, we went back and examined the ICD codes false positives (FP) and false negatives (FN). We noted that of the 39 FP and the 2 FN based on the manual review, the CNN correctly classified 5 of the FP and 1 of the FN, further showing the value of the deep learning models, at least in those cases misclassified by ICD coders. We have revised the discussion section accordingly.

**[New] REVIEWER COMMENT on Model Validation:** The authors have responded to the concern raised by both reviewers about lack of *model* validation (cf process validation) by citing (a) funding limitations, and (b) challenges of data sharing. I do not understand why data sharing is relevant -- you simply need to share the *trained model* from one hospital, and apply it to the other data. This does not require sharing any data itself. You have access to and have already prepared both data sets; it should be straightforward to take a trained model and apply it to the test data from the other hospital. This is far less time-consuming or costly than the manual analysis that you have added.

**AUTHOR RESPONSE:** Although the reviewer is mostly correct, the problem is a little more nuanced than that. In addition to sharing the trained model, you would also have to share the tokenizer, which may include some protected health information.

**[New] REVIEWER COMMENT on prior work:** I appreciate the added paragraph on page 4 with prior work; individual cited work should have the relevant citation numbers added there, e.g. "Cusick and colleagues (2021)^35". Note, however, that here and elsewhere (e.g. p3) it is not meaningful to compare AUC for different task framing and different input data; the inherent difficulty of prediction will be very different from that of detection, and the difficulty of detection will vary depending on how much data is available (e.g. a single ED triage note cf. 6 months of clinical notes). The added statement "the predictive model's performance (especially as highlighted with the AUC) is fairly competitive with what has been previously reported in the literature using different approaches with EHR data, including structured data." really isn't meaningful without more explicit clarification of comparibility of the task settings.

**AUTHOR RESPONSE:** We thank the reviewer for the insightful comment and fair critique. To that end, we add the recommended explicit clarification.

**[New] REVIEWER COMMENT on index event:** "Notes recorded within 24 hours of the index event" -- does this mean 24 hours before or after the index event? I assume before, given how the data was collected, but this should be clarified.

**AUTHOR RESPONSE:** We thank the reviewer for pointing this out. The timestamp of the index event refers to the timestamp of the ICD code for a given visit. So “within the 24 hours” period refers to +/- 24hrs, given that timestamp of ICD code assignments for a given admission typically falls within that time frame of a clinical note. We have added that clarification.

**REVIEWER RESPONSE to Author response "the replication of the prior methodology is significant as this is a core tenant of the scientific method."** I humbly submit that in the context of data-driven methods, the authors' argument here about scientific method is not the key concern. What you are measuring in this work is not the inherent validity of the method, which requires some extrinsic truth to hold. You are measuring the stability of the method as applied in two different data contexts. However, if the method is biased, and the data selection methods are biased, two similar results only reflect consistency in the bias, not evidence that the method is "valid". The similar performance of the method in these two distinct data contexts may be a result of the process itself, not the inherent correctness of the method.

Neither the original work nor this replication are applied prospectively under the condition of real-world data distribution. Rather, both are evaluated only with highly controlled (and similarly biased) selection of cases and controls. The authors have stated as justification for doing this "However, at a single institution, we do not have sufficient data to develop and test the models in a real-world environment." -- yet, they have down-selected controls substantially (although arguably even at 5:1 it would not reflect the true distribution) so they could use this to test in a less biased data set.

Note also you do have to focus on replication rather than reproduction, given that you are not reproducing the methodology on the same dataset. Please review your terminology. As the other reviewer has highlighted as well, given that your focus is on replicating a *process* rather than the study itself this should be clearly identified. This is distinct even from the typical discussions of reproducibility vs replicability of models or their findings (see e.g. <https://doi.org/10.1001/jama.2019.20866> )

**AUTHOR RESPONSE:** We thank the reviewer for this insightful comment and for recommending the paper by Beam, Manrai, and Ghassemi (2020), which provided valuable guidance in refining our terminology and framing. We have addressed this issue in the detailed response to the first comment.

# REVIEWER #3

**REVIEWER COMMENT:** Authors have greatly improved the manuscript and this is rigorous and sensical work!

**AUTHOR RESPONSE:** We thank the reviewer for their suggestions for improving our manuscript.

**REVIEWER COMMENT:** Regarding the ICD Validation through Manual Review on page 12, and the implications. Previous work has shown that ICD codes are very poor at identifying psychiatric conditions, including suicidality, so it was surprising to see only 2 false negatives. How do the authors explain this unusual finding and what does it mean for the generalizability of the results? If ICD codes have such a high recall in particular, why even the need to develop alternative methods using unstructured data?

**AUTHOR RESPONSE:** We thank the reviewers for this insightful comment and agree about this being a surprising finding and one of the reasons for looking at another institution for replicability. The authors would like to point out that a very specific set of ICD codes were used, namely those defined by the Centers for Disease Control and Prevention’s National Health Statistics Report referenced in the Participants section of the Methods, and does not represent general mental health ICD codes. We have included some discussion about the implications in the Discussion section as well as some discussion about the value of models leveraging unstructured data when comparing the performance of the ICDs vs. CNNs. Specifically, upon closer examination of the false positives and false negatives by ICD as compared to manual review, we noted that the CNN classification was correct in 5 of the 39 ICD false positives and 1 of the 2 ICD false negatives, which suggests that the CNN could provide more precise retrieval of cases, e.g., for surveillance tasks, beyond simple ICD queries.

**REVIEWER COMMENT:** Page 3, last paragraph. When discussing the current approaches to suicide risk assessment... Healthcare professionals administering clinical interviews, and then documented in the notes and structured data (and clinical decision making such as to admit or send to ED, etc.) is exactly what the EHR data is from. The existence of EHR data means that encounter happened, and happened well. For example, page 4, "abundance of valuable and relevant information within the EHRs" -- that is put there by clinicians after a clinical assessment. From reading the discussion, seems that not having to *repeat* the clinical interview/questionnaires in different treatment settings (or highlight in more in the chart) after it's already been done... that's the utility! That could be explicit in the intro & discussion.

**AUTHOR RESPONSE:** We agree completely and thank the reviewer for emphasizing the utility of reducing repeated administration of clinical interviews and questionnaires across multiple treatment settings. We have specified this point in the Introduction and Discussion sections as recommended.

We would like to thank the reviewers for their diligent critiques and thoughtful comments. We hope that we have addressed the reviewers’ comments and suggested edits. We believe these edits have further strengthened our submission. We are excited to share this work with the Plos ONE readership and the rest of the mental health research community.

# REFERENCES:

Beam et al 2020. <https://pmc.ncbi.nlm.nih.gov/articles/PMC7335677/>
